# Tailored approaches to stroke health education (TASHE): study protocol for a randomized controlled trial

**DOI:** 10.1186/s13063-015-0703-4

**Published:** 2015-04-19

**Authors:** Joseph Ravenell, Ellyn Leighton-Herrmann, Amparo Abel-Bey, Alexandra DeSorbo, Jeanne Teresi, Lenfis Valdez, Madeleine Gordillo, William Gerin, Michael Hecht, Mildred Ramirez, James Noble, Elizabeth Cohn, Giardin Jean-Louis, Tanya Spruill, Salina Waddy, Gbenga Ogedegbe, Olajide Williams

**Affiliations:** Department of Population Health, NYU School of Medicine, 227 E. 30th Street NY, New York, NY 1001 USA; Department of Neurology, Columbia University Medical Center, 710 West 168th Street, New York, NY 10032 USA; Research Division - The Hebrew Home at Riverdale, 5901 Palisade Avenue, Bronx, NY 10471 USA; The Pennsylvania State University, 208 Biobehavioral Health Building University Park, Pennsylvania, PA 16802 USA; The Pennsylvania State University, 501 Keller Building University Park, Pennsylvania, PA 16802 USA; Center for Health Innovation, Adelphi University, P.O. Box 701, Garden City, NY 11530 USA; National Institute of Neurological Disorders and Stroke at the National Institutes of Health, 6001 Executive Boulevard, North Bethesda, MD 20852 USA

**Keywords:** Stroke, Community-based research, Narrative persuasion, Health disparities, Randomized trial, Stroke health education, Stroke action test

## Abstract

**Background:**

Stroke is a leading cause of adult disability and mortality. Intravenous thrombolysis can minimize disability when patients present to the emergency department for treatment within the 3 − 4½ h of symptom onset. Blacks and Hispanics are more likely to die and suffer disability from stroke than whites, due in part to delayed hospital arrival and ineligibility for intravenous thrombolysis for acute stroke. Low stroke literacy (poor knowledge of stroke symptoms and when to call 911) among Blacks and Hispanics compared to whites may contribute to disparities in acute stroke treatment and outcomes. Improving stroke literacy may be a critical step along the pathway to reducing stroke disparities. The aim of the current study is to test a novel intervention to increase stroke literacy in minority populations in New York City.

**Design and Methods:**

In a two-arm cluster randomized trial, we will evaluate the effectiveness of two culturally tailored stroke education films – one in English and one in Spanish – on changing behavioral intent to call 911 for suspected stroke, compared to usual care. These films will target knowledge of stroke symptoms, the *range of severity* of symptoms and the therapeutic benefit of calling 911, as well as address barriers to timely presentation to the hospital. Given the success of previous church-based programs targeting behavior change in minority populations, this trial will be conducted with 250 congregants across 14 churches (125 intervention; 125 control). Our proposed outcomes are (1) recognition of stroke symptoms and (2) behavioral intent to call 911 for suspected stroke, measured using the Stroke Action Test at the 6-month and 1-year follow-up.

**Discussion:**

This is the first randomized trial of a church-placed narrative intervention to improve stroke outcomes in urban Black and Hispanic populations. A film intervention has the potential to make a significant public health impact, as film is a highly scalable and disseminable medium. Since there is at least one church in almost every neighborhood in the USA, churches have the ability and reach to play an important role in the dissemination and translation of stroke prevention programs in minority communities.

**Trial registration:**

NCT01909271; July 22, 2013

## Background

### Introduction

Stroke is the leading cause of adult disability, with an estimated 795,000 new and recurrent strokes occurring annually in the US [1,2]. Stroke is also among the most economically burdensome conditions, costing US taxpayers > $60 billion annually [1,2]. Furthermore, stroke is a significant contributor to health inequities. In comparison to Whites, Blacks are at least twice as likely to die from stroke, and they have a four-fold higher incidence of first-ever stroke, particularly among those aged 34-55 years [3].

The administration of intravenous thrombolytic therapy with tissue plasminogen activator (t-PA) following an ischemic stroke can reduce the patient’s disability by 31-50% [4]. However, the narrow 3 − 4½ h treatment window is a critical limitation on the effectiveness of treatment with t-PA [4,5]. Currently, only 3-5% of diagnosed ischemic stroke events receive t-PA therapy. This low rate of treatment is predominantly due to the public’s inability to identify and respond appropriately to stroke symptoms when they occur [6]. Stroke education studies have shown that placing an emphasis on calling 911 for suspected stroke can lead to reduced pre-hospital delays [4] and increased thrombolysis for acute stroke [7].

The cascade of events and behavioral responses that occur in the pre-hospital setting of acute stroke include: (1) the *onset* of stroke-like symptoms, (2) the *recognition* of those symptoms by the patient, family or observer, (3) the *perception* of stroke-like symptoms as symptoms that warrant immediate medical attention, (4) the decision to seek medical attention, and (5) the choice of transportation to the emergency department (ED) (i.e., calling 911 for an ambulance versus an alternative mode of transportation) [8]. The *onset* of symptoms may not always correspond to the *recognition* of the symptoms, with the former influencing the consideration of t-PA therapy and the latter influencing the likelihood of seeking medical attention.

In a review of 182 stroke education studies, we found that there is a discrepancy between knowledge concerning stroke symptoms and the reaction to an acute manifestation of one or more of these symptoms [9]. Mass media campaigns have had some success in increasing the public’s knowledge of stroke symptoms, but have provided little evidence that such knowledge is related to help-seeking behaviors [10]. At the core of the problem is the relationship among three elements, (1) the recognition of any one cardinal stroke symptom, (2) the perceived severity of the symptom, and (3) the ambiguity of the situation that may determine one’s action – to call or not to call 911. At high levels of perceived severity, the specific recognition of a particular symptom is less important than at low levels. For example, when one experiences weakness in one side of his body, such that he is no longer able to stand up, or witnesses such in another person, the situation is unambiguous and a call to 911 is clearly warranted. In contrast, when a symptom is perceived to have low severity or transiency (e.g., mild focal weakness, numbness or transient monocular blindness), ambiguity is greater and the likelihood of calling 911 is diminished. This remains the case even if that person is aware that such deficits can be a stroke symptom [11]. However, it is recommended that the recognition of a symptom, even at a very low level of perceived severity and the resultant ambiguity, result in a call to 911.

### Stroke disparities

Despite higher stroke incidence rates compared to Whites, Blacks and Hispanics are less likely to use ambulance services, which is associated with delayed arrival at the ED and ineligibility for intravenous thrombolysis for acute stroke [12]. This delay in immediate presentation in the ED is largely dependent on patients’ recognition of stroke symptoms and their urgency [5]. In previous studies, our group has found very low stroke literacy rates – which we define as knowledge of stroke symptoms and when to call 911 for suspected stroke – among Blacks and Hispanics compared to Whites [13-17]. These findings may, in part, be responsible for disparities in acute stroke treatment rates [11]. Increasing stroke literacy may be an effective means for improving recognition of symptoms and the need for an urgent response. This latter component – emphasis on *urgent action* in response to stroke symptoms (i.e., calling 911 and getting to the hospital) – is a critical component of stroke symptom recognition required for appropriate action that may increase use of thrombolytic therapy [9]. Thus, novel interventions that address the *perception of severity of symptoms as urgent* – a subtlety that has been often overlooked by previous stroke educational interventions – are needed.

### Narrative persuasion

**Figure 1:**
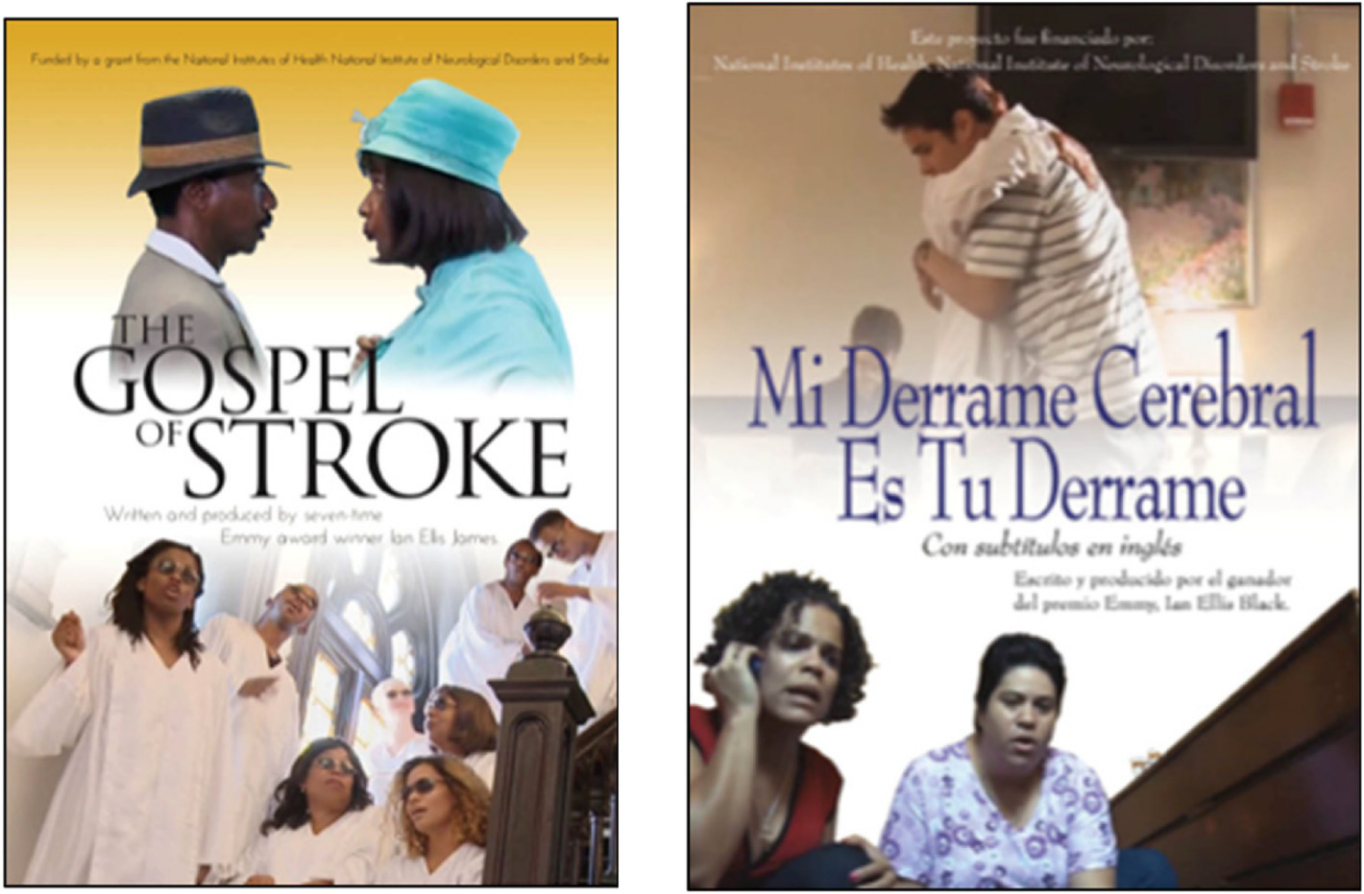
English and Spanish short stroke films.

In this study, we will build on our previous work that identified barriers to increasing stroke literacy and behavioral intent to call 911 for acute stroke [11,18]. With a focus on Black and Hispanic populations in New York City, we developed and pilot-tested a novel, culturally tailored intervention that uses storytelling and narrative persuasion to increase stroke literacy, in the form of two professionally produced films – one in English and one in Spanish (Figure 1).

Narrative approaches, which include stories, drama, personal experience and the experience of others, may be especially effective among populations with a strong oral tradition [19]. Early Black Americans learned about their culture, their contributions to human civilization and strategies for surviving adversity through the lens of orally handed down stories [20]. Building on this tradition, the use of culturally tailored narrative approaches to health communication may be a novel method for stroke-related health communication [21]. Narrative persuasion has been shown to lower blood pressure in African Americans with hypertension [22] and improve decision-making regarding prostate cancer screening of patients with low health literacy [23]. Narrative approaches may also play a significant role in health behavior interventions targeting Hispanics. Spanish-language video narratives known as *telenovelas* have been shown to increase breast cancer knowledge, positively influence attitudes toward breast cancer screening [24], positively influence attitudes toward substance use prevention [25], and increase HIV awareness and testing [26,27].

In this study, Tailored Approaches to Stroke Health Education (TASHE), we will evaluate the effectiveness of our narrative film intervention on increasing stroke literacy in 250 individuals (125 intervention; 125 control) from 14 churches at the 6-month and 1-year follow-up. Churches are among the most credible institutions within most communities and play a particularly influential role in African-American and most Hispanic cultures. They also provide access to groups that convene regularly and are a valuable delivery channel for health promotion programs. Additionally, oral narratives are a critical component of religious communication, making church congregants a potentially receptive audience for experiential storytelling [28]. Several studies have supported a church-based approach to health behavior interventions for Black and Hispanic communities, including stroke reduction in at-risk populations [29-39].

## Methods

### Study design overview

TASHE is a two-phase study design. In the first phase, we developed two 12-min, culturally tailored, theatrical films about stroke – English and Spanish versions. The films were designed to increase stroke literacy, with a focus on behavioral intent to call 911. The second phase is a two-arm cluster randomized trial in which we will evaluate the effectiveness of the intervention films, compared to the usual practice (Usual Care) of distributing stroke education brochures to lay people, in improving stroke literacy in churches located in Black and Hispanic communities. This study has received ethics approval from the Columbia University Medical Center Institutional Review Board (IRB-AAAK5853).

### Community action plan

A Community Advisory Committee (CAC) of church pastors, other key church personnel and stroke survivors was established to work with an overarching transdisciplinary group that includes the study leadership, a health communication expert and the film producer, for the purpose of assisting with tailoring the stroke film production to meet the spiritual, cultural and educational needs of their congregations. In addition to helping with the development of the intervention, the CAC will meet with the study staff to discuss and provide ongoing feedback on the delivery of the intervention throughout the course of the study. Once analyses are completed, the process evaluation and outcomes will be reviewed with the CAC to garner their opinions on the meaning of the findings for their community and co-develop mechanisms for dissemination.

### Film development

Five focus groups conducted with stroke survivors (*N* = 31) in New York City helped inform film development. Three groups were conducted in English and two in Spanish. The focus groups were held in churches and community centers that were centrally located and handicapped accessible. Two experienced researchers facilitated the groups using a semi-structured, open-ended interview guide. Individual follow-up interviews were conducted with select participants when the story seemed to have more depth that could be explored or when further clarity was needed.

The focus groups and interviews were recorded and transcribed verbatim. Data were analyzed using content analysis techniques [40] and coded using Atlas.TI 7.1.8. Themes were identified, and storyboards that reflected the voices and stories of the respective communities were produced. An advisory committee reviewed the storyboards and provided additional comments and suggestions. The themes, comments and suggestions were incorporated into the films, which told the real stories of stroke as seen and felt by the community members.

### Church setting

In 2004, the Office of Minority Health of the NYC Department of Health formed the Borough of Brooklyn Ecumenical Advisory Group (BBEAG), comprised of 506 faith-based organizations (FBO) in Brooklyn. In 2006, the BBEAG formed the Borough of Manhattan Ecumenical Advisory Group (BMEAG), whose membership includes 100 FBOs. The mission of both groups is three-fold: (1) to serve as an advocate and conduit to eliminate and prevent health disparities, (2) to promote wellness to the residents of Manhattan and Brooklyn through health education and health outreach, and (3) to broaden health care access and services in partnership with the Office of Minority Health. These ecumenical networks provided support for the acceptance and promotion of the intervention and the recruitment of 14 study churches from our target communities.

### Church champions

Community involvement and ownership are important elements of a successful and sustainable intervention. As such, we will also recruit one member of each congregation to serve as the project church champion. Church champions will work with the RAs to schedule recruitment and study implementation events at the church. Church champions will also deliver informal stroke film screenings to all congregants following the completion of the 1-year formal data collection period. Church champions will receive $60 for their overall assistance and participation. To show our appreciation as well as keep them engaged in the project, the research team will also plan small social events or occasional gifts for the champions and other church administration that provide assistance with the implementation of the TASHE study.

### Study procedures

#### Church recruitment and randomization

Recruitment will occur first at the church level, then the participant level. Randomization will occur at the church level to prevent contamination and other threats to internal validity through interactions between members of the same church. Once a church agrees to participate, signs a covenant of agreement and identifies a church champion, they will be randomized to either the intervention or UC arm. The randomization sequence will be generated by the study statistician at a site away from the churches, in accordance with the CONSORT guidelines.

To be included in the study, churches must have at least 250 active members (we defined “active” as members who attend church weekly). The 14 churches will be randomly assigned to either the intervention (*N* = 7) or the usual care (*N* = 7) conditions, with 25 participants recruited and enrolled at each church. We estimate the prevalence of traditional stroke risk factors (such as hypertension) in these churches to pattern that of NYC population, which is 38% among Blacks and 30% among Hispanics, based on the 2012 NYC Department of Health Community Health Survey. In the event that fewer eligible participants are identified at specific churches than we anticipate, additional churches will be added as necessary. Each church will receive a $1000 donation as compensation for their time and space needed during the study period.

### Participant recruitment

Participants will be recruited from the church congregation. Following randomization of the churches, church personnel will assist the RA in organizing recruitment events after church services, where the research team will present the study and explains the rationale, significance and procedures. The RA will coordinate with the champion to determine space and convenient schedules for recruitment events as well as participant interviews. RAs will host blood pressure screening events where interested potential participants can have their blood pressure taken and learn more about the study. If interested, potential participants will receive an explanation of the study. Informed consent will be obtained from each participant. Participants will then be contacted the following week to schedule their eligibility screening and baseline interview.

Potential participants must meet certain inclusion criteria in order to be eligible for participation. Inclusion criteria include: (1) no prior history of stroke, (2) high risk – defined as a history of one or more stroke risk factors: hypertension, diabetes, tobacco use, abdominal obesity, heart disease and/or high cholesterol, (3) aged 34 years or older (due to the large increase in stroke incidence among minority groups at age 34 [41]), (4) self-identifies as Black or Hispanic, (5) a regular congregant of the church (attends services at least twice a month), and (6) lives in a household with a telephone. Exclusion criteria include: (1) unable to give consent, (2) has a participation-prohibiting disability, (3) history of dementia, (4) terminal illness or other medical illness resulting in mortality in less than 1 year, (5) severe hearing impairment, and (6) planning to move out of NYC in the next 3 years.

### Retention

To address this critical issue, we will request the names, addresses and telephone numbers of three friends or relatives who would know how to get in touch with the participant. We request this information during the consent process and have found this approach helpful for retention in our other trials. We will implement additional strategies that have led to successful recruitment and retention of participants in clinical trials, such as: (1) making regular telephone contact to remind participants of upcoming study appointments and update their contact information, (2) providing a toll-free study telephone number to contact study staff and report a change of address or telephone number, (3) having an ethnically diverse staff from similar backgrounds as participants, and (4) providing a small stipend after study visits to compensate participants for their time and reimburse transportation costs [42-50].

The research team will stress the importance of compliance with the study sessions at enrollment. Participants will receive $30 to cover time and transportation for their baseline interview visit and $15 at each subsequent visit – the church screening for intervention participants, and the 6-month and 1-year follow-ups for participants in both groups ($75 intervention; $60 control). RAs will make reminder telephone calls to participants on the day prior to each interview session. Finally, RAs will be flexible in accommodating participants’ schedules, so they can be contacted evenings and/or weekends.

### Data and safety monitoring plan

To ensure the safety of participants and the validity and integrity of the data, a data and safety monitoring board (DSMB) has been established and meets approximately every 6 months. The DSMB includes senior investigators with expertise in stroke, hypertension and biostatistics. Adverse events will be reported to the appropriate IRBs and Privacy Boards.

### Study intervention

#### Intervention condition

It is widely accepted in the health promotion literature that interventions are more effective when they are culturally appropriate [51]. Thus, we optimize the potential impact of the intervention by enhancing its cultural appropriateness through cultural targeting. Using a transdisciplinary approach, we produced two culturally tailored, 12-min films (English and Spanish versions) in the first phase of the study. To accomplish this critical component, we organized a team of cultural leaders from local Black and Hispanic churches, a stroke expert, community-based researchers with expertise in culturally appropriate interventions, behavioral scientists – including narrative communication experts – a specialist in theory of knowledge acquisition, and a professional filmmaker. The director of the film is a seven-time Emmy award winning writer and producer of entertainment media who has experience in stroke narrative productions. To address the major barriers in the cascade of events and behavioral responses that occur in the pre-hospital setting of acute stroke, the screenplay has five didactic goals: (1) communicate knowledge of stroke symptoms, (2) communicate the *range of severity* with which the symptom may occur – for example, a slight slurring of the speech should be enough to precipitate action, (3) communicate the therapeutic benefit of calling 911, (4) overcome barriers to calling 911, including the reluctance to go to the ED, and (5) overcome issues related to denial or embarrassment if symptoms turn out not to be serious.

At baseline, the RA will provide participants with a study letter and the stoke film DVD (either in Spanish or in English) and ask them to watch it at least twice at home. The RA will also invite them to the church for one formal, assembly-style screening of the film. The RA will coordinate with the church champion to determine space and convenient schedules for the group film viewing. Participants will be reminded of the time and location of the film screening beforehand via phone. If a participant is unable to attend the larger group film screening, the RA will schedule an individual screening at a time and place more convenient for the participant.

### Viewing protocol

The number of exposures to the stroke films was selected based on theories governing “effective frequency” from marketing studies [52]. “Effective frequency” is defined as the number of exposures to an advertising message required for achieving effective communication and influencing consumer purchasing behavior. It is generally accepted that a single exposure, if relevant to the recipient’s concerns, may achieve effective communication. However, the marketing literature suggests that repeated exposure will be more effective and a minimum of three exposures may be required [52].

### Control condition

Educational brochures in English and Spanish are the most common form of stroke education. This practice occurs within inpatient, outpatient and community settings (particularly health fairs), and will represent “usual care” for the purpose of our study. The most common source of materials is from national organizations, such as the American Stroke Association (ASA), National Stroke Association (NSA) and National Institutes of Neurological Diseases and Stroke (NINDS). As such, an educational brochure will represent the control condition. For consistency, we will utilize one from the NINDS (in both English and Spanish) describing the five cardinal stroke symptoms and the importance of calling 911. The brochure will be distributed to control participants at the end of the baseline interview. There will be only one distribution of educational materials to the control group. Control participants will be asked to read over the brochure at least two times.

### Treatment fidelity

Assessment of fidelity will be based on the expanded Lichenstein treatment fidelity model developed by the OBSSR Behavior Change Consortium [53]. Similar to other DVD-based interventions [22,54], we will employ the following strategies to assess the dose of the intervention received by subjects: (1) all subjects will sign an attendance sheet to verify that they attended the DVD viewing in the church, (2) immediately following the in-church film screening, participants will complete the Stroke Action Test (STAT) measure, and (3) participants will also complete a Narrative Performance Scale (NPS). To minimize cross-contamination – some subjects may attend more than one church, or may know people from other participating churches – we will inform those exposed to the film not to divulge information about the film.

### Data collection and management

This study has a Research Core that is experienced in coordinating clinical trials and will be responsible for the following activities: (1) development of the computer-assisted data collection system, (2) staff training and certification in data collection, (3) randomization procedures, (4) data monitoring and quality control, (5) data processing, and (6) data analysis. The Research Core will prepare regular reports for internal and external monitoring of progress toward study milestones and provide blinded and unblinded data requests for DSMB meetings.

### Eligibility screening and baseline interview

The eligibility screening and baseline interview will be the same for both conditions. The RA will administer the eligibility screening and, if the participant is eligible, the baseline measure to all subjects via an electronic survey conducted on a laptop computer. This visit will be conducted at the church in a private location to ensure privacy. The eligibility screening will take approximately 30 min to complete, and the baseline screening will take approximately 45-60 min.

### Intervention film screening

After the in-church, assembly-style film screening, participants will complete the STAT and the NPS instruments. The STAT is a validated instrument that uses hypothetical scenarios to assess behavioral intent to call 911. The NPS is a validated instrument that assesses the extent to which participants are “transported” into the world of the narrative and became engaged with the story line and its characters [55].

### Follow-up interviews

Follow-up interviews will be the same for both conditions. The assessment will be administered in-person to study subjects using a structured interview format at 6 months and 1 year post-intervention. For the intervention group, 6 months will be measured from the date of the in-church screening. For the control group, 6 months will be measured from the date of the baseline interview. If after multiple attempts the RA is unable to schedule and complete the follow-up interview in-person, the participant will be given the option to complete the interview via telephone. If the staff is unable to reach a participant to complete the 6-month follow-up, the staff will still attempt to contact him/her for the 1-year follow-up.

### Church champion interviews

Following the initial year of formal data collection, annual interviews will be conducted with the church champion until year 5 of the study. This interview will assess the number of stroke film screenings conducted at the church after the formal 1-year study period.

### Study measures

#### Behavioral intent to call 911

The primary outcome will be self-reported behavioral intent to call 911 based on the validated STAT instrument [56]. This measure reliably predicts the actions that respondents would take if a stroke occurs and serves as our measure of stroke literacy. Most stroke knowledge assessments use either recall tasks (spontaneously naming stroke symptoms) or recognition tasks (selecting the correct stroke symptoms from a list), but the situation of acute stroke is different. During an acute stroke situation, symptoms are *experienced* or *observed.* The identification of symptoms is meaningless if it does not lead to urgent action. Knowing to call 911 when a stroke diagnosis is provided is not the same as calling 911 when stroke symptoms present.

The STAT measures behavioral intent to call 911 for suspected stroke symptoms using hypothetical stroke scenarios in which the stroke diagnosis is not provided. Each scenario features one of the cardinal stroke symptoms or a distracter symptom, and respondents are asked how they would respond to the scenario. The five cardinal signs of stroke include weakness, speech impairment, vision impairment, headache and dizziness [57]. The 21 scenarios are derived from these 5 cardinal “suddens” stroke symptoms. There are 21 because the 5 cardinal symptoms are presented in a variety of situations. All participants in the intervention and control conditions will be administered the STAT at baseline and the 6-month and 1-year follow-ups.

### Stroke knowledge

Following the STAT survey, we will directly assess knowledge of the five cardinal “suddens” stroke symptoms, which we will present in a multiple-choice format alongside seven distracter symptoms derived from the STAT. A composite score will be calculated by counting the number of correct responses to the five stroke symptoms [57] and seven distracter symptoms (maximum score = 12), modeled after the Behavioral Risk Factor Surveillance System (BRFSS) [58]. Participants will be administered the stroke knowledge battery at baseline and the 6-month and 1-year follow-ups.

### Demographics

At baseline, we will collect demographic information, such as age, sex, race, educational status and income. We also collect the following modules of the NINDS Common Data Elements (CDE) related to stroke: demographics, social status, medical history, family history, medication history and behavioral history. All time-varying demographic variables will also be collected at the 6-month and 1-year follow-up interviews (e.g., physical activity, diet, health, etc.).

### Sample size and power analysis

#### Power calculations for the primary outcome (behavioral intent to call 911 using the stroke action test)

Power calculations were based on both examination of endpoint differences and on rates of change over time using data from all three waves of data collection. The main calculation shown in Table 1 is for the primary analysis examining rate of change. The outcome is treated continuously, and we assumed a two-tailed test with α = 0.05 and 1-β = 0.80. The following assumptions, based on the literature on stroke literacy and stroke action using the STAT [56], were used to calculate sample size and power: R =0.85 (reliability); g = 2 (group); σ = 17.83 (pooled SD of examinee subgroups); δ = 8, 9, 10 (Stroke Action point reduction); d = δ/σ where δ is the endpoint mean differences, and d is Cohen’s d (0.45, 0.50, 0.56). The number of churches posited to be economically feasible is 7 per arm, and the cluster size is 18, with over-recruitment to 25. The ICC derived from data from over 30 churches participating in a separate, ongoing randomized trial of a church-based intervention was posited to be 0.02. Thus, the VIF = 1+ (clustersize-1)*ICC = 1.34. It was assumed that ρ = 0.6 (the average correlation between baseline and follow-up).

**Table 1:** **Sample size (** ***n*** **per group) for different effect sizes with power >80%**

	**δ = 8 (d = 0.45)**	**δ = 9 (d = 0.50)**	**δ = 10 (d = 0.56)**
ρ = 0.6	79	62	50
ρ = 0.6	δ = 8.19 (d = 0.47)	δ = 7.10 (d = 0.40)	δ = 6.34 (d = 0.36)
	75	100	125

### Power for rate of change on the stroke action test

Using formulas based on Diggle et al. [59], assuming three time points (baseline, 6 months and 1 year), the requisite sample sizes are shown in Table 1. It was determined, based on both sets of calculations, that 125 subjects per group will provide >80% power to detect an 8-point change in STAT. Lower effect sizes, about 6 points, can also be detected based on testing the Time X Group interaction in a mixed model, which allows for heterogeneous variances, clustering, unreliability and serial correlations.

### Power calculation for the secondary outcome (stroke symptom knowledge)

For this binary outcome, it is posited that the control group will improve by 6%, starting at 4% with knowledge, based on a composite score for correct answers to the five cardinal “suddens” symptoms [57]. Given randomization, the intervention group is posited to start at the same level and improve 24%, for a net improvement of 18%. The assumptions are: R =0.85 (reliability); g = 2 (group); cluster size = 18; ICC = 0.02; VIF = 1+ (clustersize-1)*ICC = 1.34. A sample size of 125 subjects per group will provide 80% power to detect a group difference in the rate of increased knowledge at 6 months, from a baseline rate of 4% to a desired level of 28%.

### Data analytic plan

#### Primary hypothesis

*Participants who receive the culturally tailored narrative film intervention will demonstrate greater behavioral intent to call 911 for suspected stroke, as measured by the STAT at immediate post-intervention, 6 months and 1 year following the intervention compared to those in the usual care arm.* The change from pre- to post-intervention values of continuous outcomes (STAT scores) will be modeled as functions of treatment group, time, and the interaction of time and treatment. The primary test concerns the Group × Time interaction, and the resulting F-test will provide the primary “intent-to-treat” test of the hypothesis. The magnitude of the treatment effect, with 95% CI, for STAT scores will be estimated. Design effects of clustering within churches and repeated measures together with unreliability, serial correlations and possible heterogeneous variances will be modeled when necessary.

### Secondary hypothesis

*Participants who receive the culturally tailored narrative film intervention will demonstrate greater stroke symptom recognition at 6 months and 1 year following the intervention compared to those in the usual care arm.* Binary outcomes (stroke symptom knowledge) will be examined using generalized estimating equations (GEEs) with a logit link. Because randomization is at the church level, with clustering, such models allow for modeling the correlation due to clustering and repeated measures. Stroke knowledge will be coded as knowledgeable or not, based on predetermined cut scores. Baseline knowledge will be included in the model. Results can be converted to odds ratios. In the event that the above described logit analyses indicate one or more sources of potential bias, the predicted values of those analyses will be included as covariates in the a multilevel logistic regression model (SAS, version 9).

Prior to analyses, baseline values of all variables from each arm will be examined; however, no *p* values will be provided, and covariates are not proposed for inclusion in the main analyses of treatment effects. If one or more sources of potential (selection or attrition) bias are identified, the predicted values from those analyses will be included as covariates in secondary analyses. Depending on the severity of missing data, other modeling techniques may be used.

## Discussion

Stroke is the leading cause of adult disability in the US. However, disability from stroke can be mitigated by treatment with intravenous thrombolysis among patients arriving to the ED within the 3 − 4½ h therapeutic window [4,5]. Unfortunately, timely stroke treatment with thrombolysis, which can increase the odds of minimal to zero disability at 3-months by 31% to 50%, has been severely limited because of pre-hospital delays, especially among minority groups [5-7,60]. The overarching goal of the proposed intervention is to reduce stroke disparities by overcoming pre-hospital barriers related to emergency stroke treatment and facilitating the appropriate response to the acute stroke situation using a novel, culturally tailored approach developed by an experienced, transdisciplinary team.

To our knowledge, this is the first randomized trial of a culturally tailored narrative media intervention to improve stroke outcomes among urban Black and Hispanic adult churchgoers. Many pastors believe health and spirituality are linked, and are interested in participating in health-related programs [28]. Disease prevention programs targeting healthy behaviors have been evaluated in churches, including increased fruit and vegetable intake, adoption of physical activity, weight loss and smoking cessation [29-38], and have been proven successful in many cases. Churches are a valuable and effective delivery channel for behavior change programs, including stroke education [39]. This is particularly true for minority groups, given their historically low participation in clinical trials. Furthermore, oral narratives are a big component of religious communication. Experiential storytelling is a cornerstone of sermons and has been used by myriad denominations to inspire congregations.

Much of the published faith-based research focuses on African Americans and the Black church. As such, additional research is needed to test the effectiveness of church-based interventions among Hispanics, a much more heterogeneous group. Findings suggest that approximately 90% of Hispanics identify with a particular religion, and most Hispanics believe “that religion is very important in their lives” [61], which supports the inclusion of more church-based health behavior interventions in research. Several studies have supported a faith-based approach to health behavior interventions for Hispanics communities [36-38]. One such example is the Stroke Health and Risk Education (SHARE) study, which found a substantial burden of stroke risk factors among Mexican-American (the largest subgroup of Hispanic Americans) church communities and suggested that church-based interventions may be a way to reduce stroke in these at-risk populations [35].

In summary, we will conduct a novel church-based trial to intervene on at-risk Blacks and Hispanics in New York City. If successful, this narrative film intervention has the potential to make a significant public health impact. Film is a highly scalable method that may represent an untapped medium for health behavior change among low literacy populations with strong oral traditions. Since there is at least one church in almost every neighborhood in the US [62,63], churches have the ability and reach to play an important role in the dissemination and translation of stroke prevention programs in minority communities. Thus, wide-spread implementation in urban communities that, like New York City, have large populations of underserved minorities who would benefit from stroke prevention interventions is an attainable goal.

## Trial status

Recruitment for this study began in December 2013 and is ongoing.

## Abbreviations

TASHE: Tailored Approaches to Stroke Health Education
BBEAG: Borough of Brooklyn Ecumenical Advisory Group
FBO: Faith-based organization
BMEAG: Manhattan Ecumenical Advisory Group
RA: Research assistant
STAT: Stroke Action Test
NPS: Narrative Performance Scale

## References

[1] Scott PA, Silbergleit R (2006). Economic benefit of increasing utilization of intravenous tissue plasminogen activator for acute ischemic stroke in the United States. Stroke.

[2] Stansbury JP, Jia H, Williams LS, Vogel WB, Duncan PW (2005). Ethnic disparities in stroke: epidemiology, acute care, and postacute outcomes. Stroke.

[3] Go AS, Mozaffarian D, Roger VL, Benjamin EJ, Berry JD, Blaha MJ (2014). Heart disease and stroke statistics–2014 update: a report from the American Heart Association. Circulation.

[4] The National Institute of Neurological Disorders and Stroke rt-PA stroke study group (1995). Tissue plasminogen activator for acute ischemic stroke. N Engl J Med.

[5] Hacke W, Kaste M, Fieschi C, Toni D, Lesaffre E, von Kummer R (1995). Intravenous thrombolysis with recombinant tissue plasminogen activator for acute hemispheric stroke. The European cooperative acute stroke study (ECASS). JAMA.

[6] Gillum LA, Johnston SC (2001). Characteristics of academic medical centers and ischemic stroke outcomes. Stroke.

[7] California Acute Stroke Pilot Registry (CASPR) Investigators (2005). Prioritizing interventions to improve rates of thrombolysis for ischemic stroke. Neurology.

[8] Muller-Nordhorn J, Wegscheider K, Nolte CH, Jungehulsing GJ, Rossnagel K, Reich A (2009). Population-based intervention to reduce prehospital delays in patients with cerebrovascular events. Arch Intern Med.

[9] Teuschl Y, Brainin M (2010). Stroke education: discrepancies among factors influencing prehospital delay and stroke knowledge. Int J Stroke.

[10] Lecouturier J, Rodgers H, Murtagh MJ, White M, Ford GA, Thomson RG (2010). Systematic review of mass media interventions designed to improve public recognition of stroke symptoms, emergency response and early treatment. BMC Public Health.

[11] Willey JZ, Williams O, Boden-Albala B (2009). Stroke literacy in central Harlem a high-risk stroke population. Neurology.

[12] Lacy CR, Suh DC, Bueno M, Kostis JB (2001). Delay in presentation and evaluation for acute stroke: stroke time registry for outcomes knowledge and epidemiology (STROKE). Stroke.

[13] Williams O, DeSorbo A, Noble J (2012). Hip Hop Stroke: The standalone effect of musical cartoons on stroke knowledge of fourth grade children living in a low-income neighborhood.

[14] Williams O, DeSorbo A, Noble J, Gerin W (2012). Child-mediated stroke communication: findings from Hip Hop Stroke. Stroke.

[15] Williams O, Noble JM (2008). ‘Hip-hop’ stroke: a stroke educational program for elementary school children living in a high-risk community. Stroke.

[16] Williams O, DeSorbo A, Sawyer V (2011). Effect of a novel intervention on caloric and menu board literacy among low-income preadolescent schoolchildren.

[17] Williams O, DeSorbo A, Noble J, Shafer M, Gerin W (2012). Long-term learning of stroke knowledge among low income children in a high-risk community. Neurology.

[18] Abel-Bey A, DeSorbo A, Valdez L, Cohn E, Williams O (2014). Barriers to calling 911 for acute stroke among minority women.

[19] Miller-Day M, Hecht ML (2013). Narrative means to preventative ends: a narrative engagement framework for designing prevention interventions. Health Commun.

[20] Hinyard LJ, Kreuter MW (2007). Using narrative communication as a tool for health behavior change: a conceptual, theoretical, and empirical overview. Health Educ Behav.

[21] Hecht ML, Krieger JK (2006). The principle of cultural grounding in school-based substance use prevention: the drug resistance strategies project. J Lang Soc Psychol.

[22] Houston TK, Allison JJ, Sussman M, Horn W, Holt CL, Trobaugh J (2011). Culturally appropriate storytelling to improve blood pressure: a randomized trial. Ann Intern Med.

[23] Volk RJ, Jibaja-Weiss ML, Hawley ST, Kneuper S, Spann SJ, Miles BJ (2008). Entertainment education for prostate cancer screening: a randomized trial among primary care patients with low health literacy. Patient Educ Couns.

[24] Wilkin HA, Valente TW, Murphy S, Cody MJ, Huang G, Beck V (2007). Does entertainment-education work with Latinos in the United States? Identification and the effects of a telenovela breast cancer storyline. J Health Commun.

[25] Kelly KJ, Stanley LR, Comello ML, Gonzalez GR (2006). Tobacco counter-advertisements aimed at bicultural Mexican American youth: the impact of language and theme. J Health Commun.

[26] O’Donnell L, San Doval A, Duran R, O’Donnell CR (1995). The effectiveness of video-based interventions in promoting condom acquisition among STD clinic patients. Sex Transm Dis.

[27] Olshefsky AM, Zive MM, Scolari R, Promoting ZM, HIV (2007). risk awareness and testing in Latinos living on the U.S.-Mexico border: the Tu No Me conoces social marketing campaign. AIDS Educ Prev.

[28] National Heart, Lung, and Blood Institute - Office of Prevention, Education and Control. Working with religious congregations: a guide for health professionals. In Book Working with religious congregations: A guide for health professionals (National Institutes of Health). Washington, DC; 1997.

[29] Campbell MK, Hudson MA, Resnicow K, Blakeney N, Paxton A, Baskin M (2007). Church-based health promotion interventions: evidence and lessons learned. Annu Rev Public Health.

[30] Kim KH, Linnan L, Campbell MK, Brooks C, Koenig HG, Wiesen C (2008). The WORD (Wholeness, Oneness, Righteousness, Deliverance): a faith-based weight-loss program utilizing a community-based participatory research approach. Health Educ Behav.

[31] McNabb W, Quinn M, Kerver J, Cook S, Karrison T (1997). The PATHWAYS church-based weight loss program for urban African-American women at risk for diabetes. Diabetes Care.

[32] Resnicow K, Campbell MK, Carr C (2004). Body and soul. A dietary intervention conducted through African American churches. Am J Prev Med.

[33] Resnicow K, Jackson A, Wang T (2001). A motivational interviewing intervention to increase fruit and vegetable intake through Black churches: results of the Eat for Life trial. Am J Public Health.

[34] Voorhees CC, Stillman FA, Swank RT, Heagerty PJ, Levine DM, Becker DM (1996). Heart, body, and soul: impact of church-based smoking cessation interventions on readiness to quit. Prev Med.

[35] Zahuranec DB, Morgenstern LB, Garcia NM, Conley KM, Lisabeth LD, Rank GS (2008). Stroke health and risk education (SHARE) pilot project: feasibility and need for church-based stroke health promotion in a bi-ethnic community. Stroke.

[36] Bopp M, Fallon EA, Marquez DX (2011). A faith-based physical activity intervention for Latinos: outcomes and lessons. Am J Health Promot.

[37] Martinez SM, Arredondo EM, Perez G, Baquero B (2009). Individual, social, and environmental barriers to and facilitators of physical activity among Latinas living in San Diego county: focus group results. Fam Community Health.

[38] Peak T, Gast J, Ahlstrom D (2010). A needs assessment of Latino men’s health concerns. Am J Mens Health.

[39] Boden-Albala B, Edwards DF, Clair SS, Wing JJ, Fernandez S, Gibbons MC (2014). Methodology for a community-based stroke preparedness intervention. The Acute Stroke Program of Interventions Addressing Racial and Ethnic Disparities Study. Stroke.

[40] Krippendorff KH (2013). Content analysis: an introduction to its methodology.

[41] Kleindorfer D, Broderick J, Khoury J, Flaherty M, Woo D, Alwell K (2006). The unchanging incidence and case-fatality of stroke in the 1990s: a population-based study. Stroke.

[42] Yancey AK, McCarthy WJ, Harrison GG, Wong WK, Siegel JM, Leslie J (2006). Challenges in improving fitness: results of a community-based, randomized, controlled lifestyle change intervention. J Womens Health (Larchmt).

[43] Yancey AK, Miles OL, McCarthy WJ, Sandoval G, Hill J, Leslie JJ (2001). Differential response to targeted recruitment strategies to fitness promotion research by African-American women of varying body mass index. Ethn Dis.

[44] Yancey AK, Ortega AN, Kumanyika SK (2006). Effective recruitment and retention of minority research participants. Annu Rev Public Health.

[45] Russell C, Palmer JR, Adams-Campbell LL, Rosenberg L (2001). Follow-up of a large cohort of Black women. Am J Epidemiol.

[46] Janson SL, Alioto ME, Boushey HA (2001). Asthma Clinical Trials N: attrition and retention of ethnically diverse subjects in a multicenter randomized controlled research trial. Control Clin Trials.

[47] Staffileno BA, Coke LA (2006). Recruiting and retaining young, sedentary, hypertension-prone African American women in a physical activity intervention study. J Cardiovasc Nurs.

[48] Vollmer WM, Svetkey LP, Appel LJ, Obarzanek E, Reams P, Kennedy B (1998). Recruitment and retention of minority participants in the DASH controlled feeding trial. DASH collaborative research group. Dietary approaches to stop hypertension. Ethn Dis.

[49] Moorman PG, Newman B, Millikan RC, Tse CK, Sandler DP (1999). Participation rates in a case-control study: the impact of age, race, and race of interviewer. Ann Epidemiol.

[50] Wiemann CM, Chacko MR, Tucker JC, Velasquez MM, Smith PB, DiClemente RJ (2005). Enhancing recruitment and retention of minority young women in community-based clinical research. J Pediatr Adolesc Gynecol.

[51] Kreuter MW, Lukwago SN, Bucholtz RD, Clark EM, Sanders-Thompson V (2003). Achieving cultural appropriateness in health promotion programs: targeted and tailored approaches. Health Edu Behav.

[52] Krugman HE (1972). Why three exposures may be enough. J Advert Res.

[53] Bellg AJ, Borrelli B, Resnick B, Hecht J, Minicucci DS, Ory M (2004). Treatment fidelity workgroup of the NIHBCC: enhancing treatment fidelity in health behavior change studies: best practices and recommendations from the NIH behavior change consortium. Health Psychol.

[54] Kreuter MW, Holmes K, Alcaraz K, Kalesan B, Rath S, Richert M (2010). Comparing narrative and informational videos to increase mammography in low-income African American women. Patient Educ Couns.

[55] Larkey LK, Hecht M (2010). A model of effects of narrative as culture-centric health promotion. J Health Commun.

[56] Billings-Gagliardi S, Mazor KM (2005). Development and validation of the stroke action test. Stroke.

[57] Signs and symptoms of stroke [http://www.stroke.org/understand-stroke/recognizing-stroke/signs-and-symptoms-stroke]

[58] Greenlund KJ, Neff LJ, Zheng ZJ, Keenan NL, Giles WH, Ayala CA (2003). Low public recognition of major stroke symptoms. Am J Prev Med.

[59] Liang DPJ, K-Y ZSL (1994). Analysis of Longitudinal Data.

[60] Morgenstern LB, Staub L, Chan W, Wein TH, Bartholomew LK, King M (2002). Improving delivery of acute stroke therapy: the TLL temple foundation stroke project. Stroke.

[61] Changing faiths: Latinos and the transformation of American religion [http://pewhispanic.org/reports/report.php?ReportID=75]

[62] Hoffman MS (1988). 1987 Yearbook of American and Canadian Churches.

[63] Lincoln CE. Knowing the Black Church: What it is and why? In: The State of Black America. National Urban League; 1989: 137-50

